# Tissue transglutaminase induces Epithelial-Mesenchymal-Transition and the acquisition of stem cell like characteristics in colorectal cancer cells

**DOI:** 10.18632/oncotarget.15370

**Published:** 2017-02-16

**Authors:** Oluseyi Ayinde, Zhuo Wang, Martin Griffin

**Affiliations:** ^1^ School of Life and Health Sciences, Aston University, Aston Triangle, Birmingham B4 7ET, United Kingdom

**Keywords:** tissue transglutaminase, Epithelial-Mesenchymal Transition, cancer stem cells, colorectal cancer

## Abstract

Human colon cancer cell lines (CRCs) RKO, SW480 and SW620 were investigated for TG2 involvement in tumour advancement and aggression. TG2 expression correlated with tumour advancement and expression of markers of epithelial-mesenchymal transition (EMT). The metastatic cell line SW620 showed high TG2 expression compared to the primary tumour cell lines SW480 and RKO and could form tumour spheroids under non- adherent conditions. TG2 manipulation in the CRCs by shRNA or TG2 transduction confirmed the relationship between TG2 and EMT. TGFβ1 expression in CRC cells, and its level in the cell medium and extracellular matrix was increased in primary tumour CRCs overexpressing TG2 and could regulate TG2 expression and EMT by both canonical (RKO) and non-canonical (RKO and SW480) signalling. TGFβ1 regulation was not observed in the metastatic SW620 cell line, but TG2 knockdown or inhibition in SW620 reversed EMT. In SW620, TG2 expression and EMT was associated with increased presence of nuclear β-catenin which could be mediated by association of TG2 with the Wnt signalling co-receptor LRP5. TG2 inhibition/knockdown increased interaction between β-catenin and ubiquitin shown by co-immunoprecipitation, suggesting that TG2 could be important in β-catenin regulation. β-Catenin and TG2 was also upregulated in SW620 spheroid cells enriched with cancer stem cell marker CD44 and TG2 inhibition/knockdown reduced the spheroid forming potential of SW620 cells. Our data suggests that TG2 could hold both prognostic and therapeutic significance in colon cancer.

## INTRODUCTION

Colorectal cancer (CRC) is the third most common cancer in the world and a major cause of morbidity and mortality [1]. Although advances have been made in the treatment of CRC over the last decade with the introduction of new surgical techniques, radiotherapy and chemotherapy, the overall survival rate of patients with CRC has not shown a marked improvement [2]. Survival of the disease is highly dependent upon the stage of disease at diagnosis, and typically ranges from a 90% 5-year survival rate for cancers detected at the localized stage, 70% for regional, 10% for people diagnosed for distant metastatic cancer [3]. Metastasis plays a critical role in the poor prognosis, and more than one-third of patients with CRC will ultimately develop metastatic disease [4].

The bulk of carcinoma cells generally exhibit a predominance of epithelial characteristics. However, in order to invade, disseminate to distant tissues and subsequently form metastatic colonies, neoplastic epithelial cells must shift, at least transiently, into a more mesenchymal phenotype. Epithelial-Mesenchymal-Transition (EMT) is a physiological process found in embryonic development, tissue remodelling and wound healing [5]. In neoplasms it is a critical step in the progression of tumour cells from in situ carcinoma to distant metastasis. Furthermore, recent reports suggest that EMT results in the acquisition of other properties involved in carcinoma progression, such as increased resistance to apoptosis, drug resistance, increased motility and the acquisition of stem cell-like properties [6].

The multifunctional enzyme tissue transglutaminase (TG2) is a protein involved in a number of cellular roles, including the post translational modification of proteins, as a scaffold protein in cell adhesion and as a cell signalling protein [7–9]. TG2 also acts as an important part of a pro-inflammatory response and has been associated with EMT in both fibrosis and cancer [10]. TG2 is associated with various physiological and pathological conditions [11]. Clinical studies have correlated TG2 expression with metastatic cancer and poor survival outcomes of ovarian, breast and colon cancer patients [12, 13]. It is also suggested that TG2 mediates several aspects of cancer cell behaviour, including motility, invasion, growth, and survival [11, 14, 15]. Although recent studies in different cancer cell types suggest a role for TG2 in EMT as highlighted by a possible cross talk between TG2 and three critical pathways in EMT, e.g. Transforming Growth Factor β 1 (TGFβ1), Wnt, β-catenin and Nuclear factor kappa light chain enhancer of activated B (NFκB) [16, 17], there still remain a number of conflicting reports on the importance of TG2 in cancer progression with respect to its pro and/or anti-cancer roles [18–22]. This may in part be accounted for by the pleiotropic nature of TG2 owing to its multifunctional roles [19].

Using the well characterised human colorectal cancer cell lines RKO, SW480 and SW620 as a well-validated model [23] for the *in vitro* study of colorectal cancer progression we show that TG2 expression correlates with disease progression. We also show that knockdown or inhibition of TG2 results in the reduced ability of CRCs to acquire a mesenchymal and stem cell like phenotype. We also show, dependent on the cell line, that TG2 plays an important role in multiple pathways in the induction of EMT.

## RESULTS

### TG2 expression correlates with disease progression in this CRC model

**Figure 1:**
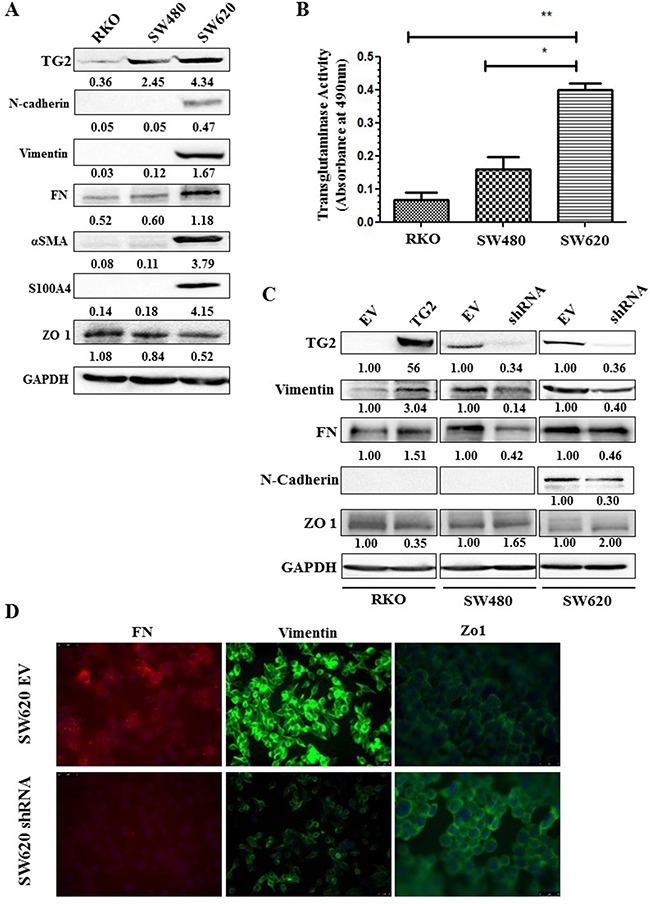
TG2 expresssion correlates with disease progression and EMT **A**. Western blotting of whole cell lysates from wt RKO, SW480 and SW620 cells showing expression of TG2, EMT and disease severity markers. SDS-PAGE and Western blotting were carried out as described in the Materials and Methods. **B**. TG2 activity measured in whole cell lysates of wt RKO, SW480 and SW620 cells undertaken as described in the Materials and Methods. *p<0.05*, *, significant from SW480, **, significant from RKO. Data are represented as mean ± S.D, (n=3). **C**. Western blotting for TG2 and EMT markers in CRCs with TG2 expression increased by viral transduction (TG2) or reduced by transduction with TG2 shRNA (SW480shRNA and SW620shRNA) and their corresponding transduced empty vector (EV) controls. **D**. Immunofluorescent detection of TG2 and EMT marker epitopes by fluroscence microscopy in TG2 manipulated cells and their corresponding controls (EV). Representative image from two independent experiment.

TG2 expression was determined in cell lysates of three well characterised colon cancer cell lines RKO, SW480 and SW620, via Western blotting. RKO and SW480 are primary human CRC cell lines, while SW620 is a lymph metastatic cell line. SW480 and SW620 are an isogenic pair obtained from the same patient and serve as an *in vitro* model for tumour progression [23]. Figure 1A shows that TG2 expression was increased in the metastatic cell line SW620 compared to the two primary cancer cell lines SW480 and RKO with more TG2 expressed in SW480 compared to RKO cells. This difference in TG2 expression followed a similar trend when levels TG2 activity were measured in the different cell lines (Figure 1B).

### TG2 is required for EMT in this CRC model

**Figure 2:**
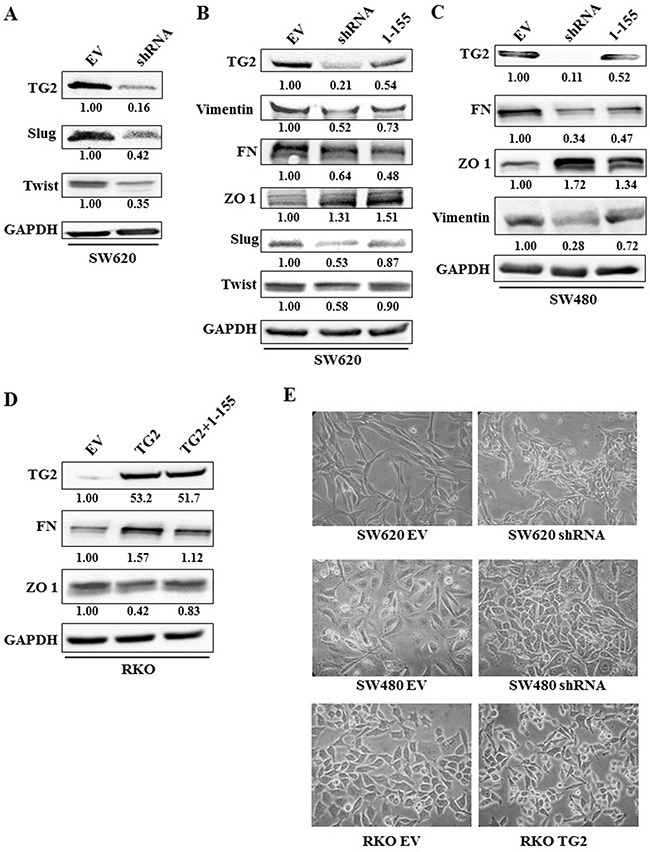
Manipulation of TG2 expression or activity correlates with EMT markers **A**. Western blot showing that TG2 expression correlates with increased expression of transcription factors of EMT(Slug and Twist) in SW620 (EV) control cells or SW620 cells transduced with TG2 shRNA (shRNA). **B**. Western blot of whole cell lysates from SW620 control cells and SW620 cells transduced with TG2 shRNA or control EV cells treated with TG2 selective inhibitor 1-155 (1 μM). Control cells were treated with vehicle alone DMSO. **C**. Western blot of SW480 control cells and SW480 cells transduced with TG2 shRNA (SW480shRNA) or wt cells treated with TG2 selective inhibitor 1-155 (1 μM). Control EV cells were treated with vehicle alone DMSO. **D**. RKO EV cells treated with the vehicle control DMSO or TG2 selective inhibitor 1-155 (1 μM) and RKO cells transduced with TG2 (RKOTG2). **E**. Representative images of cell morphology of CRCs transduced with TG2 shRNA or wt TG2. Equal number of cells were seeded and 48 h images were taken at 40× Objective magnification.

TG2 expression was silenced in SW480 and SW620 cells by transduction of cells with TG2 shRNA. The efficiency of the different shRNA constructs on TG2 expression and corresponding effect on the expression of EMT markers are shown in Supplementary Figure 1. In RKO cells where TG2 basal levels are low, cells were transduced with the wild type TG2. Comparison of TG2 expression with the expression of EMT markers in the different cells (Figure 1C) shows that increase in TG2 expression by viral trasduction in RKO cells leads to increased expression of mesenchymal markers, including vimentin and FN, and a decrease in epithelial tight junction marker Zonal occludin 1 (ZO-1). The expression level of these markers was reversed once TG2 was downregulated by transduction of TG2 shRNA in SW480 cells. Only the metastatic SW620 cells express detectable mesenchymal markers, including N-cadherin, S100A4 and α smooth muscle actin (αSMA). TG2 downregulation by shRNA leads to reduced mesenchymal markers FN, vimentin and N-cadherin and restored levels of ZO-1 (Figure 1C). These changes in EMT markers with TG2 expression were validated in SW620 cell by immunofluorescence staining of Vimentin, Fibronectin (FN) and ZO-1 (Figure 1D). We next investigated the impact of TG2 on the upstream events of EMT by determining the level of expression of the transcription factors Slug and Twist1. Figure 2A shows that knock down of TG2 in the high TG2 expressing SW620 cells results in a significant decrease in both Slug and Twist, suggesting that TG2's role in EMT is upstream of these two transcription factors in the EMT process.

Treatment of SW620 and SW480 (Figure 2B and 2C) with the TG2 selective inhibitor 1-155 [24–26] also reduced the expression of TG2 and EMT markers— vimentin and FN in SW480 and SW620 cells, and the expression of transcription factors Slug and Twist 1 in SW620 cells. 1-155 treatment also enhanced expression of the epithelial marker ZO-1 in both cell lines. Similarly, in the TG2 transduced RKO cells, treatment with TG2 inhibitor 1-155 reduced expression of the EMT marker FN and upregulated ZO-1, compared to control TG2- transduced RKO cells, while TG2 expression by Lentiviral transduction was not affected by 1-155 treatment (Figure 2D).

Manipulating TG2 expression in RKO, SW480 and SW620 also altered the morphology of these cells (Figure 2E). Increased TG2 expression led to an elongated, fibroblast like appearance in cells, while loss of TG2 expression led to a more cuboidal, more epithelial cell like morphology.

### TGFβ1 induces TG2 and EMT in CRCs

**Figure 3:**
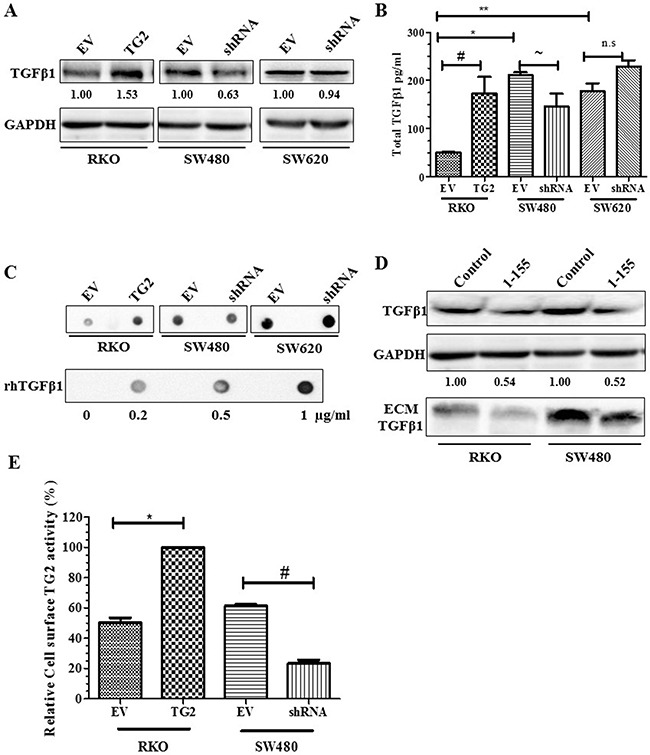
TG2 induces TGFβ1 expression and release into cell culture media **A**. Western blots of whole cell lysates of CRCs showing increased expression of TGFβ1 in whole cell lysates of RKO EV control cells, RKO transduced with TG2 (RKOTG2), SW480 or SW620 transduced with empty vectors (EV) cells or transduced with TG2 shRNA (shRNA). **B**. TGFβ1 released into cell culture media of CRCs determined by ELISA undertaken as described in the Materials and Methods. Data represent means ± S.D., from two experiments each performed in triplicate (*, **, # and ~, p<0.05). **C**. Representative Dot blotting of cell culture media of CRCs both EV controls and cell transduced with TG2 (TG2) or shRNA (SW480shRNA and SW680shRNA), and recombinant human TGFβ1 probed with TGF β1 antibody (n=3). **D**. Western blot of total TGFβ1 expression in whole cell lysate and in ECM fractions of CRCs wt RKO (treated with DMSO control) and wt SW480 cells folllowing treatment with TG2 cell permeable inhibitor 1-155 (1 μM). **E**. Cell surface TG2 activity measured by incorporation of biotin-X-cadavarine in RKO EV control cells, RKOTG2 cells, SW480 EV control cells and SW480shRNA cells measured as described in the Materials and Methods. Data are represented as mean ± S.D., (n=2), with each independent experiment performed in triplicate (* and #, p<0.05).

In CRCs, TGFβ1 signalling, an inducer of EMT, can be a major dysfunctional point during tumour progression [1]. Figure 3A shows that TGFβ1 expression correlates with TG2 expression in the whole cell lysates of RKO and SW480 when TG2 is either increased or decreased, respectively, which is comparable to the levels TGFβ1 found in the cell culture medium (Figure 3B). Moreover, TG2 inhibition by 1-155 results in reduced expression of TGFβ1 in whole cell lysates and in matrix bound TGFβ1 in primary tumour CRCs RKO and SW480 (Figure 3D). We demonstrate that the cell surface activity of TG2 (Figure 3E) in these cell lines also correlates with the presence of TGFβ1 in the extracellular milieu, suggesting a link between cell surface TG2 and Extracellular Matrix (ECM) bound TGFβ1. Interestingly, in SW620 cells, TGFβ1 levels show no correlation with TG2 expression (Figure 3B).

**Figure 4:**
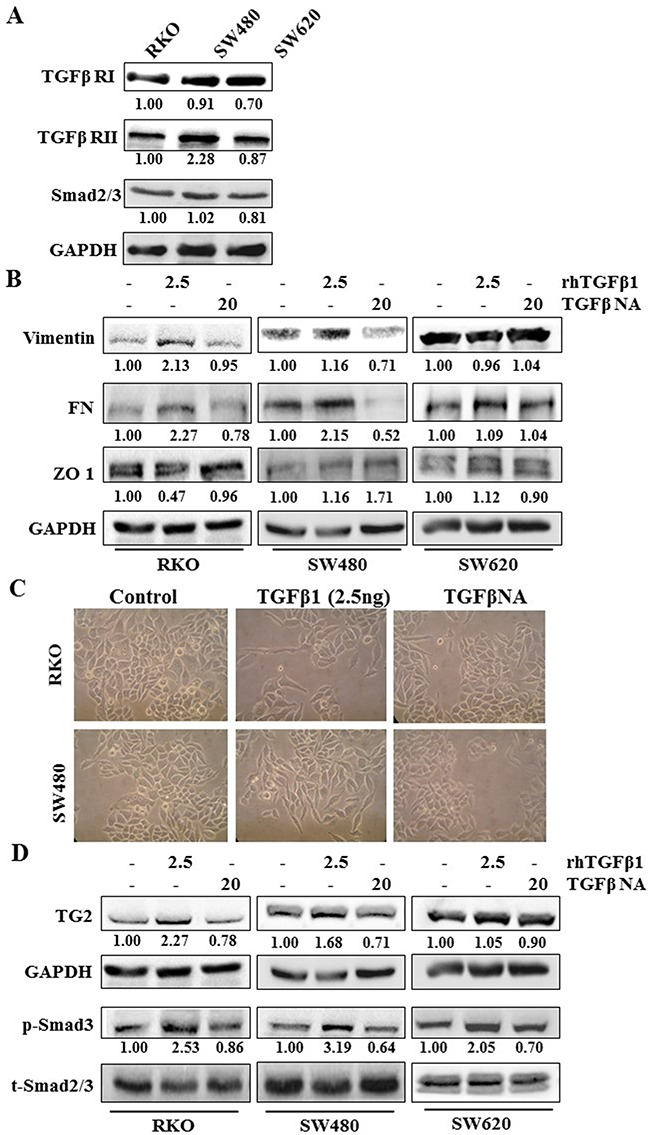
TGFβ1 induces EMT and TG2 in primary CRCs RKO and SW480 **A**. Western blotting of CRCs showing presence of TGFβ receptor (TGFβR) I and TGFβRII in wt RKO, SW480 and SW620 whole cell lysates. **B**. Western blotting of whole cell lysates of wt CRCs RKO, SW480 and SW620. Cells showing presence or absence of EMT markers following TGFβ1 treatment with and without treatment with TGFβ neutralising antibody. **C**. Representative images of cell morphology of CRCs wt RKO and SW480 treated with rhTGFβ1 (2.5 ng/ml) and rhTGFβ1 plus TGFβ1 neutralising antibody PAN (20μg/ml). Equal numbers of cells were seeded and 48 h after treatment images were taken at 20× objective using a phrase contrast microscope. **D**. Western blot of whole cell lysates from wt RKO, SW480 and SW620 cells, showing expression of TG2 after TGFβ1 treatment, with or without treatment with TGFβ neutralising antibody. CRCs were treated with human recombinant TGFβ1 (2.5 ng/ml) and TGFβ neutralising antibody (NA) (20 μg/ml) for 48 h.

All the CRCs express TGFβ1 receptor I and II (Figure 4A) and the downstream signalling molecules Smad2/3. When treated with TGFβ1, EMT was induced in the RKO and SW480 cells with increased levels of vimentin and FN and decreased ZO-1 (Figure 4B). TGFβ1 neutralising antibody treatment reduced expression of these EMT markers. The effect of TGFβ1 is confirmed by appearance of a fibroblast like phenotype in TGFβ1 treated RKO and SW480 cells (Figure 4C). Figure 4D shows TG2 expression was increased by TGFβ1 treatment in RKO and SW480 cells with a corresponding increase in the phosphorylation of Smad2/3 shown in all CRC's which could be reduced by the neutralising TGFβ antibody. However, neither TGFβ1 treatment nor TGFβ neutralizing antibody showed any effect on TG2 expression in SW620 cells.

### TGFβ1 induces EMT in primary CRCs RKO and SW480 via a non-canonical extracellular signal regulated kinase (ERK) pathway

**Figure 5:**
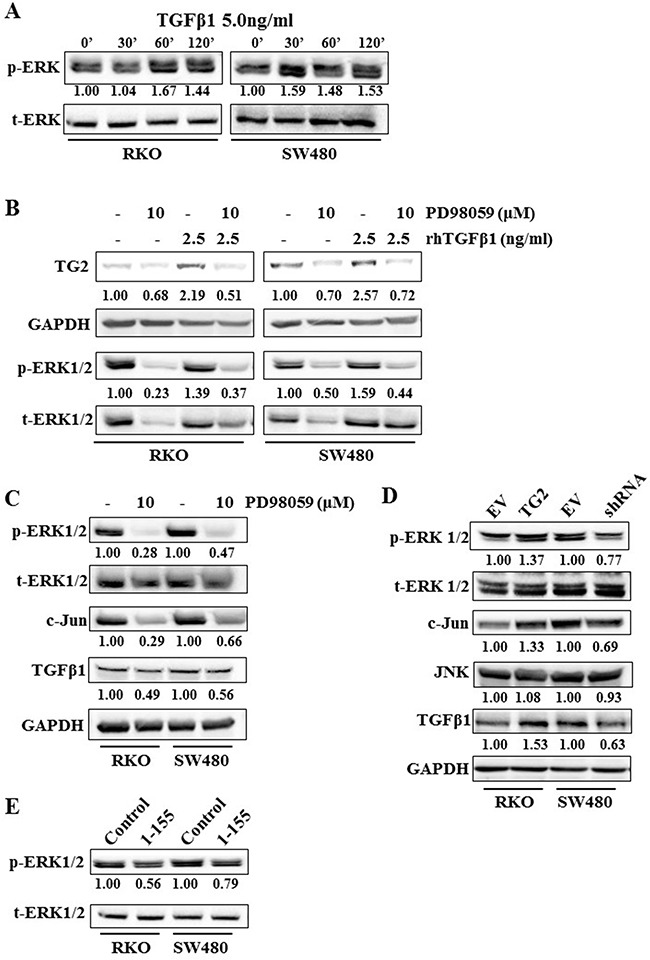
ERK1/2 plays a role in TGFβ1 induced TG2, and TG2 induced TGFβ1 expression in RKO and SW480 cells **A**. Western blotting showing ERK1/2 activation, after TGFβ1 (5.0 ng/ml) treatment over a time course of 2 h in wt RKO and SW480 cells. **B**. Western bloting of whole cell lysates of wt RKO and SW480 cells, showing expression of TG2 and ERK1/2 after treatment with TGFβ1 with or without ERK inhibitor PD98059 (10 μM). **C**. Western blotting of whole cell lysates from wt RKO and SW480 cells showing expression of ERK1/2, C-Jun and TGFβ1 with or without treatment of cells with ERK inhibitor PD98059 (10 μM). **D**. Western blotting of whole cell lysates of RKO and SW480 control cells and cells transduced with TG2 (RKOTG) or shRNA (SW480shRNA), showing TG2 expression, ERK1/2 activation and TGFβ1 expression. **E**. Western blotting of whole cell lysates showing activation of ERK1/2 in wt RKO and SW480 cells with or without treatment with TG2-sepcific cell permeable inhibitor 1-155 inhibitor (1 μM).

The data obtained for TGFβ1 with RKO and SW480 cells suggests a link between TGFβ1 signalling and TG2 expression. However, since SW480 is a Smad4 null cell line and RKO expresses wild type Smad4 [27, 28], we next studied whether a potential Smad4-independent pathway in the SW480 cells which may also play a role in RKO cells is involved in inducing TG2 expression and EMT. TGFβ1 has been shown to mediate the activation of a number of downstream targets. In both cell lines, ERK phosphorylation was increased above background between 30-120 min following TGFβ1stmulation (Figure 5A). Furthermore, inhibition of ERK1/2 by ERK1/2 inhibitor PD98059 reduced TG2 expression even in the presence of TGFβ1 when compared to the control cells (Figure 5B), suggesting that TGFβ1 may also employ the Mitogen activated protein kinase (MAPK) pathway for induction of TG2 in both RKO and SW480 cells.

It has been reported that ERK signalling can induce C-Jun [29]. In Figure 5C, we show that PD98059 reduced the expression of C-Jun with a corresponding decrease in TGFβ1 expression, indicating the involvement of C-Jun in ERK1/2-induced TGFβ1 expression in RKO and SW480 cells. We also show that knockdown (SW480) or increase (RKO) in expression of TG2, or treatment with the selective inhbitor 1-155 results in reduced phosphorylation of ERK1/2 (Figure 5D and 5E).

### TG2 induces EMT via multiple mechanisms in the different colon cancer cell lines

**Figure 6:**
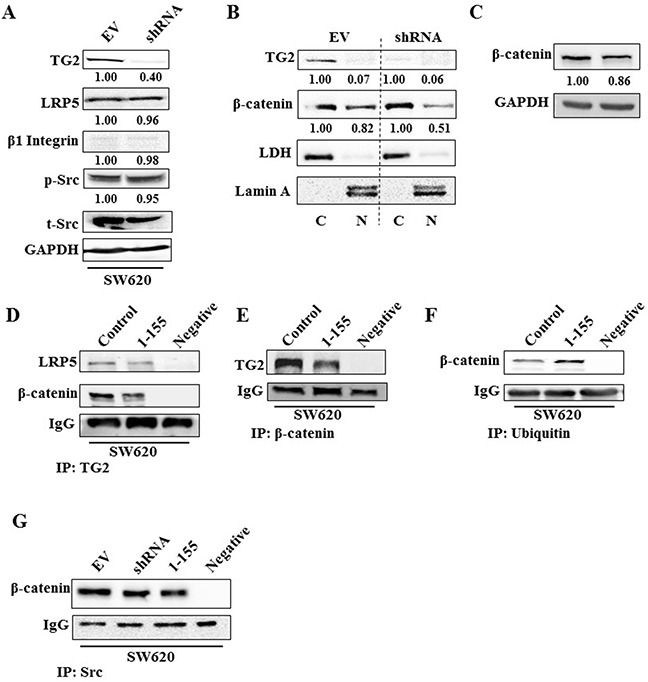
TG2 interacts with LRP5 prevents ubiquitination of β-catenin and induces β-catenin accumulation in the nucleus **A**. Western blotting of whole cell lysates of SW620 EV control cells and SW620 transduced with TG2shRNA (SW620shRNA), showing expression of TG2, LRP5, β1 Integrin, phosphorylated and total Src. **B**. Western blotting of β-catenin presence in the nuclear (N) and cytoplasmic (C) in cell extracts of SW620 control cells and SW620shRNA cells. LDH and Lamin A were used as cytoplasmic and nuclear protein markers respectively. The N and C fractions were separated as described in the Materials and Methods. **C**. Western blotting of β-catenin expression in whole cell lysates of SW620 control cells and SW620shRNA cells. **D**. Western blotting of LRP5 and β-catenin in wt SW620 treated with DMSO (control) and SW620 cells treated with TG2 selective inhibitor 1-155 (1 μM), following TG2 co-IP from whole cell lysates. PBS containing no cell lysates was used as the negative control sample. Co-IP was undertaken as described in the Materials and Methods. **E**. Western blotting of whole cell lysates for TG2 after β-catenin co-IP, following treatment of cells with TG2-selective inhibitor 1-155 (1 μM) or DMSO control. PBS containing no cell lysates was used as the negative control sample. **F**. Western blotting of whole cell lysates from wt SW620 cells after treatment with TG2 selective inhibitor 1-155 (1 μM) or DMSO control, showing β-catenin after ubiquitin co-IP of whole cell lysates from wt SW620. PBS containing no cell lysates was used as the negative control sample. **G**. Western blotting of β-catenin after Src co-IP from whole cell lysates of SW620 EV cells (treated with DMSO) and SW620 shRNA cells or after treatment with TG2 inhibitor 1-155. PBS containing no cell lysates was used as the negative control sample.

Since TGFβ1 does not impact on EMT in the metastatic SW620 cells despite TG2's ability to induce EMT in these cells (Figure 4B and 4C), we looked at the potential involvement of the Wnt/β-catenin signalling pathway and key integrins, through which TG2 could be involved in driving EMT. SW620 is rich in the TG2 binding receptor Low-density lipoprotein receptor-related protein 5 (LRP5), but shows very little expression of β1 Integrin (Figure 6A). Importantly, we show that in SW620, knock down of TG2 expression by shRNA leads to the reduction of the translocation of β-catenin into the nucleus (Figure 6B and 6C). By using co-IP, we demonstrate that TG2 interacts with β-catenin which can be reduced by the TG2 selective inhibitor 1-155 (Figure 6D and 6E). We also confirm by co-IP that TG2 binds to LRP5 (Figure 6D), a co-receptor with frizzle and LRP6 in the propagation of Wnt signalling [30]. This interaction can be reduced in cells treated with TG2 inhibitor 1-155 (Figure 6D).

Figure 6F shows via co-IP, that β-catenin interaction with ubiquitin is increased when cells are treated with TG2 inhibitor 1-155 when compared to control cells. Interestingly, in SW620 cells neither TG2 knockdown nor inhibition with 1-155 altered the interaction between β-catenin and Src (Figure 6G), suggesting that TG2 is able to inhibit ubiquitination of β-catenin, downstream of Src in SW620 cells.

### Colon cancer stem cells express TG2 and β-catenin

**Figure 7:**
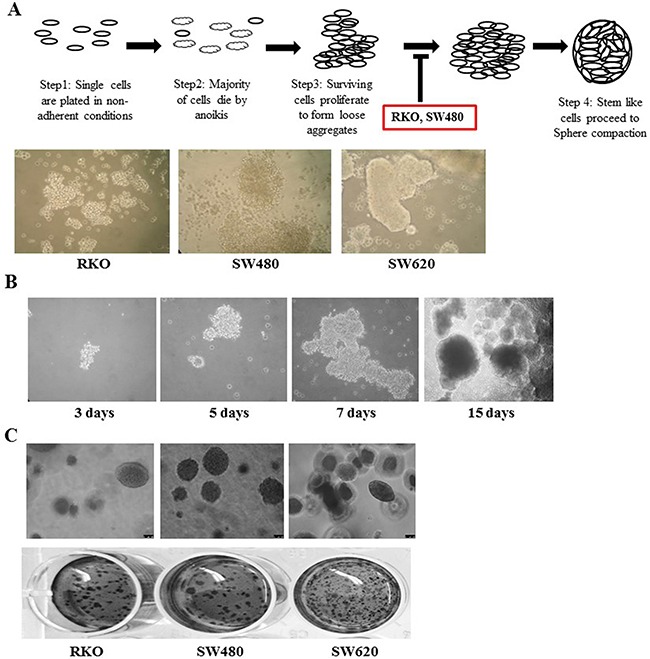
Metastatic SW620 cells form spheroids in non adherent cell culture **A**. Spheroid formation *in vitro*, wt primary cells SW480 and RKO form loose aggregates, while wt metastatic SW620 cells proceed to form compact spheroids. Representative images were taken after 12 days of culture. **B**. Stages of spheroid formation in wt SW620 cells allowed to grow for 15 days. Compaction of spheroids is observed from 5 days with the precense of a dense core in the spheroid 15 days into the culture. Spheroids formation was captured at 20× objective days 3, 5, 7 and 10× Objective Day 15. **C**. Soft Agar colony formation assay for CRCs. Colonies were allowed to grow for 14 days, and then stained with crystal violet. Representative images of colony formation on soft agar (top panel) at 10× Objective magnification.

**Figure 8:**
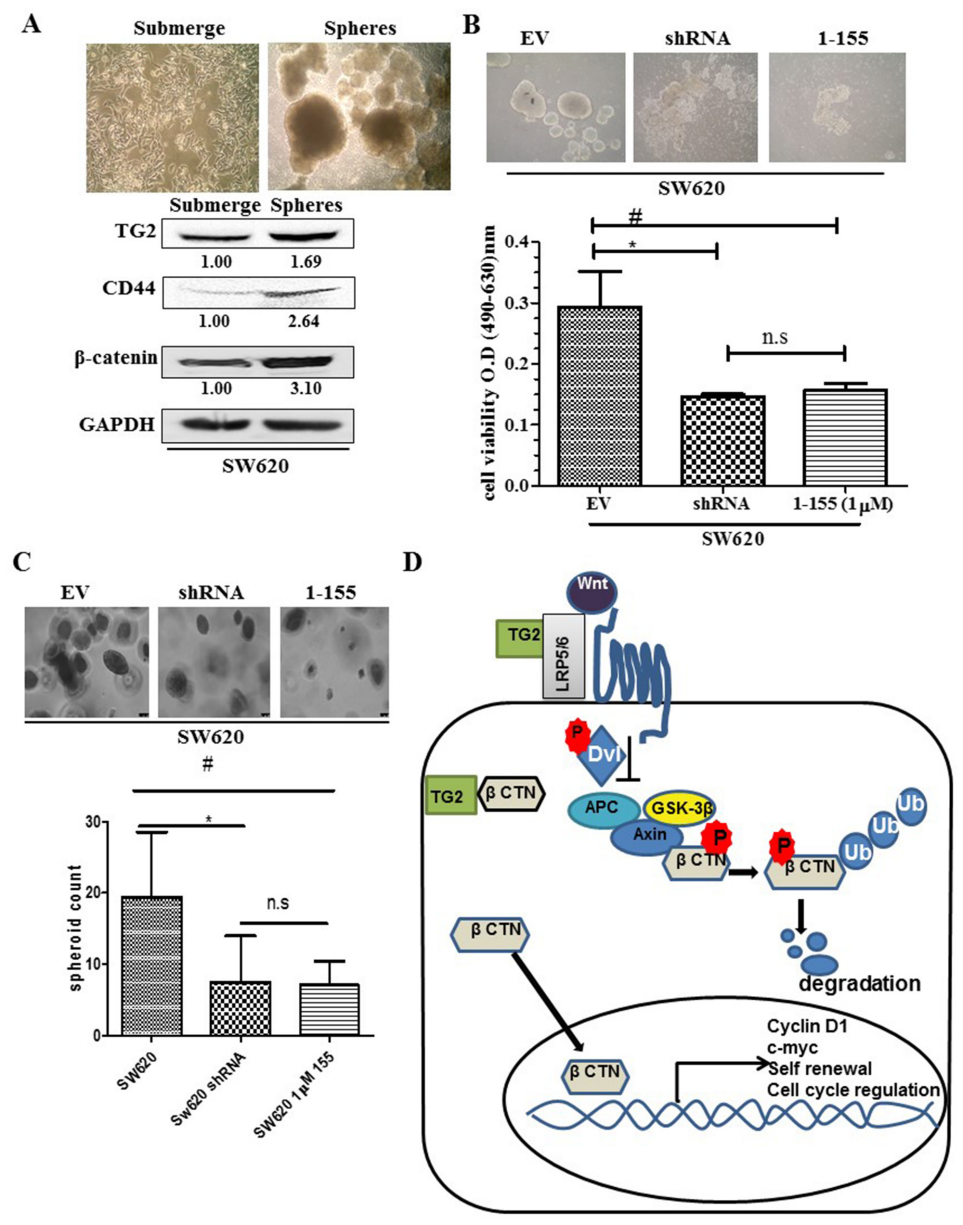
TG2 and β-catenin promotes cancer stem cell formation in CRC SW620 **A**. Representative images taken at 10× magnification of wt SW620 monolayer and SW620 spheroid cells. Western blotting of wt SW620 monolayer and spheroid cells detecting the presence of TG2, stem cell marker CD44 and β-catenin expression in whole cell lysate. **B**. TG2 knockdown (SW620shRNA) or TG2 inhibition (by 1 μM 1-155) reduces the enrichment of spheroids with stem cell like phenotype. For treatment group, CRCs were seeded in spheroid forming media contain 1 μM TG2 selective inhibitor 1-155 for 10 days, then spheroid images were captured and cell viability was performed using XTT. Data in the histogram are represented as mean ± S.D. n=3;*, # p<0.05; NS, not significant. **C**. Soft Agar formation assay for SW620 cells 14 days after seeding cells, colonies were stained with crystal violet. Colony number was determined by counting the amount of spheroids formed using a 10× objective over 10 different fields per well using a phase contrast microscope. **D**. Schematic showing extracellular TG2 interaction with LRP5 in the propagation of Wnt signalling. Cytosolic TG2 interaction with β-catenin inhibits ubiquitination of β-catenin allowing for cytosolic accumulation and nuclear translocation of β-catenin where it interacts with lef-2/TCf transcription factors to regulate cell renewal, EMT and cell cycle progression.

Unlike RKO and SW480 cells which were unable to form compact spheres, the metastatic cell line SW620 proliferates and forms compact spheroids (Figure 7A) in non-adherent cell culture. Figure 7B shows representative images of the formation of SW620 spheroids in this type of cell culture over fifteen days. Surviving cells proliferate, aggregate and eventually form compact spheroids with a dense core observed after 15 days. Given these differences in spheroid formation, we looked at the clonogenic ability of RKO, SW480 and SW620 on soft agar (Figure 7C). Metastatic SW620 cells readily formed colonies compared to primary cells SW480 and RKO. Comparison of the SW620 spheroids to the parental SW620 cells cultured in adherent conditions (Figure 8A) shows that the spheroids from SW620 cells express increased levels of the stem cell marker CD44, compared to parental cells, suggesting the spheroids are enriched with stem cell like cells. In addition, both TG2 and β-catenin are over expressed in the SW620 spheroids. We next attempted to determine the importance of TG2 in the enrichment of stem cells and clonogenicity. In Figure 8B and 8C, both TG2 knockdown and TG2-specific inhibitor 1-155 (1 μM) treatment significantly reduced the spheroid forming potential of SW620 as measured by cell viability of the spheroids. TG2 knockdown or inhibition also significantly reduced the clonogenic ability of SW620 cells.

## DISCUSSION

Our previous work using the CT26 mouse CRC cells indicated the relationship between TG2 expression, TGFβ1 and FN deposition in colon cancer [19]. In this manuscript, we assess the role of TG2 in disease progression using the 3 well-characterised human CRC cell lines RKO, SW480 and SW620. The level of TG2 expression in these cell lines is associated with disease progression from primary (RKO and SW480) to metastatic (SW620), in agreement with earlier clinical studies [12, 13]. We show that high TG2 expressing SW620 cells express low levels of the epithelial marker ZO-1, but increased levels of mesenchymal markers N-cadherin, α-SMA and protein S100A4 which is associated with metastatic disease [11]. This is in contrast to the less progressive primary tumour cells SW480 and RKO which show lower levels of TG2 expression and much lower levels of EMT markers suggesting a potential prognostic role for TG2 in colon cancer. This finding agrees with the study by Miyoshi et al. [31] and Yang et al. [32] who showed that TG2 expression was higher in colon cancer tissue compared to the corresponding normal tissue. Similarly, a recent study by Fernandez-Acenero et al. [33] showed that TG2 expression in the epithelia of colon carcinomas was significantly associated with poor patient survival, and tumour metastasis. Importantly recent work by Mouradov et al. [34] using human colorectal cancer lines has shown that CRC cell line such as these used in this study are representative of the main subtypes of primary tumours at the genomic level, validating their use as tools to investigate colorectal cancer biology and responses to new potential drugs.

EMT is important during cancer progression and is the initial step in tumour metastasis and linked to drug resistance [35–37] and the acquisition of cancer stem cell like properties [10, 17, 38]. Studies have shown that TG2 influences EMT in both cancer and fibrosis [36, 27, 39]. Here we show that knock down of TG2 expression in SW480 and SW620 cells leads to decreased expression of EMT markers and increased expression of the epithelial marker ZO-1, confirming the importance of TG2 in tumour progression via enhancing EMT. This agrees with previous studies in breast, ovarian, pancreatic, renal carcinomas and gliomas which show that TG2 plays a role in inducing EMT [8]. Our cell-permeable TG2-selective inhibitor 1-155 was able to attenuate EMT in these cells. There are two mechanisms for the impact of 1-155 on TG2, the inhibition of TG2 crosslinking activity and induction of conformational changes by locking of the enzyme into an open conformation which blocks GTP binding and alters interactions with other key proteins [24]. Therefore, it is highly possible that either or both of these effects could be responsible for inhibiting the role of TG2 in inducing EMT in these cells. The finding that TG2 inhibition reduces Slug and Twist expression in the metastatic SW620 cells also confirms that TG2 is acting upstream of these transcription factors as previously found in MCF10A and MCF12A cells [37].

Aberrations occurring in the TGFβ1 and Wnt/β-catenin signalling pathways have both been implicated in EMT and tumour progression [1, 40]. It is also well reported that TGFβ1 can specifically induce TG2 expression [26], which we have confirmed in RKO and SW480 cells. Importantly, the specific effects of TGFβ1 on TG2 induction could be blocked by a TGFβ neutralizing antibody in these two cell lines. Similarly, our results show that TGFβ1 can induce EMT in RKO and SW480, suggesting that TG2 is a downstream target of TGFβ1 and is recruited by TGFβ1 to induce EMT. However increasing the expression of TG2 in SW480 did not significantly change the expression of EMT markers and further treatment with TGFβ1 did not induce anymore EMT compared to control nontreated TG2 transduced cells (Supplementary Figure 2A and 2B). This may explain some of the findings in the metastatic SW620 where TG2 expression is much higher, and TGFβ1 does not impact EMT, suggesting a more intrinsic mechanism for TG2 that is independent of growth factors or the ECM. Hence while the interplay between TG2 and TGFβ1 may be necessary to acquire a metastatic potential, further cellular mutations may be required for a metastasis with an increased but different role for TG2 in EMT which we observe in the SW620 cells.

The mechanism of TG2 induction by TGFβ1 in cells may be varied and cell specific. TGFβ1 was reported to upregulate TG2 in ovarian cancer by upregulating the activity of NFκB [17] and via the ERK and PI3/AKT pathway in diseased pulmonary fibroblast [41]. Furthermore, genomic studies on TGFβ-regulated gene expression profiles also suggest that in the human lung cancer cell line A549, TGFβ1 induces TG2 expression by a pathway that is partially dependent on the ERK signalling pathway [42]. Hence, in this study we show that ERK1/2 a downstream signalling molecule in the Ras/MAPK kinase pathway is activated in response to TGFβ1 in both primary CRCs RKO and SW480, suggesting a potential role for ERK1/2 in TGF β1-induced TG2 expression. We also show that TG2 was associated with increased expression and levels of TGFβ1 in the whole cell lysates and culture medium respectively. This agrees with our previous studies in murine colon carcinomas that showed increased levels of TGFβ1 in the medium of TG2 transfected clones, which was inhibited by the inclusion of site-directed TG2 inhibitors in the culture medium [19]. It has been suggested by others that the MAP kinase, ERK1/2 signalling pathway may result in activation of Activator protein-1 (AP-1), a transcription factor which is critical for auto-induction of TGFβ1 [43]. Here we show that TG2 increases the activation of ERK1/2 which can be reversed by silencing TG2 or by selective TG2 inhibition using 1-155. It is, therefore, plausible to suggest that activated ERK1/2 can induce c-Jun and in turn recruit transcription factor AP-1 for increased expression of TGFβ1. This is suggested by the increased expression of c-Jun in ERK1/2 activated SW480 and TG2 transduced RKO cells. Furthermore, TG2 can increase the bio-availability of TGFβ1 in the ECM, increasing the potential for the release of active TGFβ1 from the large latent TGFβ1 binding complex [14]. Alternatively, active TGFβ1 crosslinked into the matrix might be necessary to enhance and stabilise the signalling potential of this growth factor as suggested by Griffin and colleagues [26]. Taken together these effects suggest a continuous loop and mechanism, in which TGFβ1 directly, or indirectly induces TG2 via ERK1/2, and TG2 via mediating activation of ERK1/2 recruits the necessary machinery for increased TGFβ1 expression and secretion, thus sustaining EMT.

However, a role for TGFβ in EMT was not found in the metastatic cell line SW620. We, therefore, looked for alternative pathways that might be used by TG2 in driving EMT in SW620 cells. β-catenin is a known transcriptional regulator with oncogenic activity attributed to either activating mutation or interactions with mutated β-catenin interacting proteins [44], or by canonical activation of Wnt signalling. In carcinomas, β-catenin degradation is often blocked by either loss-of-function mutations of Adenomatous Polyposis Coli (APC) or by β-catenin mutations that render β-catenin stable [45]. In SW620 cells, β-catenin is over expressed and stabilised due to mutations in APC [46]. We demonstrate that TG2 is able to interact with LRP5, a Wnt co-receptor in SW620 cells which can be reduced by the TG2-selective inhibitor 1-155 which can also lock TG2 in its inactive open conformation [35]. This suggests that TG2-LRP5 interaction may be necessary to stabilise β catenin in these cells. TG2 may also stabilise β-catenin intracellularly since SW620 cells already possess loss or mutation in APC leading to loss of the glycogen synthase kinase-APC-Axin death complex, which induces proteosomal degradation of β-catenin. We show that interaction between TG2 and β-catenin is lost on TG2 inhibition by 1-155, thus allowing β-catenin association with ubiquitin. Our results also suggest that TG2 stabilisation of β-catenin is independent of Src in SW620, since neither knockdown nor inhibition of TG2 resulted in reduced Src activation, or reduced interaction between Src and β-catenin. However, in RKO and SW480 cells, TG2 did induce increased phosphorylation of Src (Supplementary Figure 3). In ovarian carcinomas TG2 recruits Src via activation through β1-integrin and FN interaction for the stabilisation of β-catenin [44]. Interestingly, in the SW620 cells, β1-integrin was poorly expressed. Therefore, Src in our cell model may have undergone some form of mutation that has left it constitutively activated with the subsequent loss of β1-integrin, or TG2-induced activation of Src is tumour specific. TG2 may therefore play both extracellular and intracellular roles in Wnt signalling [44, 47, 48] which are key to the EMT process in the SW620 cells. Whether this is through its transamidation activity or via its GTPase or protein binding activity is not know at the moment since reaction of TG2 with 1-155 is likely to affect all these functions by inactivating the active site Cys277 and at the same time locking the enzyme into its open conformation [24]. However the absence or low expression of integrins in SW620 cells may diminish the extracellular role for TG2, as TG2-FN-integrin interraction has been reported to be essential for cell adhesion, migration and invasion in epidermoid carcinoma cell line A-431 [49].

Figure 8D shows a possible mechanism in which extracellular and intracellular TG2 may perturb β-catenin ubiquitination allowing for its cytosolic accumulation and subsequent translocation into the nucleus. Nuclear accumulation of β-catenin has been associated with cancer stem cells in colon and head and neck squamous cell carcinoma cells [50]. In a similar fashion, TG2 has been shown to facilitate a stem cell phenotype in epidermal squamous carcinoma [38], ovarian [17] and breast cancer cells [51] potentiating compact tumour spheroid formation in anchorage-independent cell culture. Our current study shows that the high TG2 expressing metastatic SW620 has the ability to form spheroids in non-adherent cell culture. TG2 knockdown or inactivation in these cells reduced this ability to form spheroids. Ectopic expression of TG2 in RKO cells resulted in increased clonogenic potential in the soft agar assay. However, it is not surprising that in RKO and SW480 cells TG2 manipulation did not lead to spheroid formation as reported previously [3].

Characterisation of the SW620 spheroids indicated TG2 was upregulated in the spheroid cells compared to cells grown in monolayer, in agreement with Cao et al. [17] in ovarian cancer cells where cells also showed increased stem cell characteristics CD44+/CD117+. The TG2 enriched SW620 spheroids had a 2-fold higher expression of CD44 when compared to monolayer cells. Additionally, SW620 spheroids exhibited increased expression of β-catenin compared to monolayers cells suggesting that TG2 and β-catenin may be necessary in cancer stem cell formation [50]. More recent studies also suggest that the self-renewal capacity of cancer stem cells in colon and head and neck cancer cells was blocked by perturbation of the interaction between β-catenin and the transcription factor TCF4 in the nucleus [52]. This supports our current notion in SW620 cells where TG2 plays a role in inhibiting β-catenin ubiquitination and thus facilitates accumulation of β-catenin in the nucleus where it can interact with TCF4, enhancing the self-renewal and enhancing cancer stem cell capacity, a process that can be perturbed by inhibiting TG2.

## CONCLUSIONS

TG2 expression correlates with CRC disease progression *in vitro*, and plays a key role in EMT, either extracellularly or intracellularly in a cell type specific context via multiple mechanisms. Importantly the TG2 selective small molecule active site directed inhibitor 1-155 developed in our group was able to attenuate TG2's capability in inducing EMT. Furthermore, spheroid containing colon stem cell like cells demonstrated high TG2 expression and irreversible inhibition of TG2 by 1-155 reduced the cancer stem cell potential of CRCs. TG2 could be both a potential prognostic marker and therapeutic target for the treatment of colon cancer. Importantly, small molecule inhibitors that selectively target TG2 as demonstrated in this manuscript could be potential therapeutic agents for colon cancer treatment.

## MATERIALS AND METHODS

### Reagents and antibodies

Peptidomimetic cell-permeable TG2-selective inhibitor 1-155 [24], and the TG2 inhibitor R283 [25] were synthesized in house. Chemicals were purchased from Sigma-Aldrich (Dorset, UK), unless stated below. The antibodies used in this work were listed in Supplementary Table 1.

### Cells and cell culture

Human colorectal cancer (CRC) cell lines RKO, SW480 and SW620, a kind gift from Dr Chris Tselepsis (University of Birmingham, Birmingham, UK), were cultured in Dulbecco's Modified Eagles Medium (DMEM) (Lonza, Cologne, Germany) containing 10% FBS (Fisher, Hempstead, UK), 1% (v/v) nonessential amino acids 100 U/ml penicillin and 100 μg/ml streptomycin, unless otherwise indicated in a humidified atmosphere with 5% CO_2_ at 37°C. Enrichment of spheroid cells with stem like characteristics was performed by culturing cells on poly-hema coated low attachment plates, cells were cultured in serum free DMEM/F12 (1:1) (Lonza, Cologne, Germany) containing 2% serum free supplement B27 (Fisher, Hempstead, UK), 20 ng/ml Epithelial growth factor (EGF), 0.4% bovine serum albumin and 4 μg/ml insulin. Spheroid cells were cultured in humidified atmosphere with 5% CO_2_ at 37°C.

CRCs were treated with the TG2 selective cell-permeable inhibitor 1-155 [26] at 1 μM. CRCs were also treated with recombinant human (rh) TGFβ1 (2.5 ng/ml) (R&D Systems, Minneapolis, UK) or mouse anti-human TGFβ neutralising antibody (R&D Systems, Minneapolis, UK). Control cells were treated with treatment vehicle DMSO or PBS. All treatments were for 48 h unless otherwise indicated.

### Lentiviral transduction

In order to ectopically express or silence TG2 in CRCs, lentiviral constructs containing wild type (wt) TG2 or different shRNA's (Sigma-Aldrich, Dorset, UK) that target human TG2 were used to transduce the CRCs and allow optimisation of TG2 knock down by Wang *et al*. [53]. Comparison of wt cells with empty vector transduced control cells indicated no differences in the expression of TG2 or EMT markers Supplementary Figure 4.

shRNA sequences: 5′-CCACCCACCATATTGTTT GAT- 3′

         5′-ACAGCAACCTTCTCATC GAGT-3′

### Western blotting

SDS-PAGE and western blotting was performed by using specific antibodies as described previously, while the membranes were re-probed with GAPDH as the loading control [53, 54]. Signals were detected using the SynGene system. Densitometry was performed using the ImageJ software. Ratios indicate band intensity normalised to GAPDH from at least 2 separate experiments.

### Co-immunoprecipitation (co-IP)

Following appropriate treatment, CRC cells were lysed in the co-IP buffer [50mM Tris-HCl containing 0.25% sodium deoxycholate, 150 mM NaCl, 0.1 mM phenylmethylsulfonyl fluoride, 1% (v/v) proteinase inhibitor cocktail and 500 μM TG inhibitor R283 as described previously] [19]. Cell lysates (600 μg) were pre-cleared by incubating with 50 μl of protein A or G-Sepharose bead slurry (GE Healthcare, Buckinghamshire, UK) at 4°C for 90 min. Following incubation with 0.5 μg of primary antibody for 90 min at 4°C, the samples were incubated with 50 μl of Protein A or G Sepharose bead slurry for 2 h at 4°C. The immunocomplex was collected in 30 μl Laemmli buffer and used for Western blotting analysis.

### Dot blotting

Total number of 2.5×10^5^ cells were seeded in complete growth media for 4 h. After which the complete growth media was replaced with ITS supplemented serum free cell culture media, cells were incubated for 16-18 h and the cell culture media was collected and the dot blotting and Western blotting for the presence of TGFβ was performed as described previously [54].

### Immunofluorescence (IF)

IF staining was performed as previously described by Wang *et al*. [53], following plating of cells on chamber slides. Fluorescent Images were visualized and captured using an epifluorescent microscope.

### TG2 activity assay

The transglutaminase activity of TG2 was measured via biotin-cadaverine incorporation into FN as described previously [11]. The incorporated biotin-cadaverine was then revealed using Extravidin-peroxidase. The development of colour was terminated using 3 N HCl and the absorbance was read at 490 nm using a Spectrafluor plate reader.

Cell surface TG activity was measured by incubating live cells (2×10^4^ /well) with 0.1 mM biotin-cadaverine in serum free medium on FN-coated wells at 37°C for 2 h as described by Jones et al. [22].

### Detection of secreted TGFβ1 by ELISA

Secreted TGFβ1 was measured by ELISA following the manufacturer's instructions (Novex™, Fisher, Hempstead, UK). Briefly, CRCs were seeded into 24-well plates and allowed to adhere for 4 h in complete medium. Complete media was replaced with growth factor-free ITS replacement medium. Following 16 h incubation with the cells, the culture medium was collected for analysis.

### Soft agar assay

The “Soft agar assay” was performed to assess colony formation and anchorage independent proliferation according to the method of Akagi et al [55]. Cultures were maintained in a humidified incubator at 37°C and 5% CO_2_ for 2 weeks before colonies were counted and photographed.

### Statistics

Data were expressed as mean ± S.D. The data shown are derived from a representative experiment undertaken in triplicate (unless otherwise stated). Comparisons among different groups were performed by analysis of variance using one-way ANOVA using the GraphPad Instat software package. Significant differences between control and treatment groups were analysed by Bonferroni's Multiple Comparison Test. Statistical significant difference between data sets was defined in the text by p < 0.05 (two-sided).

## SUPPLEMENTARY MATERIALS FIGURES AND TABLES



## Abbreviations

αSMA: α smooth muscle actin;
AP-1: Activator protein 1;
CRCs: Colorectal cancer;
ECM: Extracellular matrix;
EGF: Epithelial growth factor;
EMT: Epithelial to mesenchymal transition;
ERK: Extracellular signal regulated kinase;
FN: Fibronectin;
MAPK: Mitogen activated protein kinase;
NFκB: Nuclear factor κ- light chain enhancer of activated B cells;
PI3K: Phosphoinositide 3- kinases;
TG2: Tissue transglutaminase;
TGFβ1: Transforming growth factor β1;
TNFα: Tumour necrosis factor α;
XTT: Sodium 3′-[1-(phenylamino-carbonyl)-3,4-tetrazolium]-bis (4-methoxy-6-nitro) benzene sulfonic acid hydrate;
ZO-1: Zonal occludin 1;
wt: wild type;
rh: recombinant human;
TGFβR: TGFβ receptor;
co-IP: co-immunoprecipitation;
APC: Adenomatous Polyposis Coli;
LRP 5/6: Low-density lipoprotein receptor-related protein 5/6.

## References

[1] Lamouille S, Xu J, Derynck R (2014). Molecular mechanisms of epithelial–mesenchymal transition. Nature Review Molecular Cell Biology.

[2] Liu X, Zhang Z, Sun L, Chai N, Tang S, Jin J, Hu H, Nie Y, Wang X, Wu K, Jin H, Fan D (2011). microRNA-499-5p promotes cellular invasion and tumor metastasis in colorectal cancer by targeting FOXO4 and PDCD4. Carcinogenesis.

[3] Leng Z, Tao K, Xia Q, Tan J, Yue Z, Chen J, Xi H, Li J, Zheng H (2013). Kruppel-like factor 4 acts as an oncogene in colon cancer stem cell-enriched spheroid cells. Plos One.

[4] Zhang Z, Liu X, Feng B, Liu N, Wu Q, Han Y, Nie Y, Wu K, Shi Y, Fan D (2015). STIM1, a direct target of microRNA-185, promotes tumor metastasis and is associated with poor prognosis in colorectal cancer. Oncogene.

[5] Fan F, Samuel S, Evans KW, Lu J, Xia L, Zhou Y, Sceusi E, Tozzi F, Ye XC, Mani SA, Ellis LM (2012). Overexpression of Snail induces epithelial–mesenchymal transition and a cancer stem cell–like phenotype in human colorectal cancer cells. Cancer Medicine.

[6] Mani SA, Guo W, Liao MJ, Eaton EN, Ayyanan A, Zhou AY, Brooks M, Reinhard F, Zhang CC, Shipitsin M, Campbell LL, Polyak K, Brisken C (2008). The epithelial-mesenchymal transition generates cells with properties of stem cells. Cell.

[7] Griffin M, Casadio R, Bergamini CM (2002). Transglutaminases: nature's biological glues. Biochemical Journal.

[8] Eckert RL, Kaartinen MT, Nurminskaya M, Belkin AM, Colak G, Johnson GV, Mehta K (2014). Transglutaminase Regulation of Cell Function. Physiological Reviews.

[9] Wang Z, Griffin M (2012). TG2, a novel extracellular protein with multiple functions. Amino Acids.

[10] Agnihotri N, Kumar S, Mehta K (2013). Tissue transglutaminase as a central mediator in inflammation-induced progression of breast cancer. Breast Cancer Research.

[11] Wang Z, Griffin M (2013). The role of TG2 in regulating S100A4-mediated mammary tumour cell migration. Plos One.

[12] Hwang JY, Mangala LS, Fok JY, Lin YG, Merritt WM, Spannuth WA, Nick AM, Fiterman DJ, Vivas-Mejia PE, Deavers MT, Coleman RL, Lopez-Berestein G, Mehta K (2008). Clinical and Biological Significance of Tissue Transglutaminase in Ovarian Carcinoma. Cancer Research.

[13] Oh K, Ko E, Kim HS, Park AK, Moon HG, Noh DY, Lee DS Transglutaminase 2 facilitates the distant hematogenous metastasis of breast cancer by modulating interleukin-6 in cancer cells. Breast Cancer Res.

[14] Verderio E, Gaudry C, Gross S, Smith C, Downes S, Griffin M (1999). Regulation of cell surface tissue transglutaminase: effects on matrix storage of latent transforming growth factor-beta binding protein-1. The journal of histochemistry and cytochemistry.

[15] Kumar S, Mehta K (2013). Tissue transglutaminase, inflammation, and cancer: how intimate is the relationship?. Amino Acids.

[16] Mann AP, Verma A, Sethi G, Manavathi B, Wang H, Fok JY, Kunnumakkara AB, Kumar R, Aggarwal BB, Mehta K (2006). Overexpression of tissue transglutaminase leads to constitutive activation of nuclear factor-kappa B in cancer cells: Delineation of a novel pathway. Cancer Res.

[17] Cao L, Shao M, Schilder J, Guise T, Mohammad KS, Matei D (2012). Tissue transglutaminase links TGF-beta, epithelial to mesenchymal transition and a stem cell phenotype in ovarian cancer. Oncogene.

[18] Torricelli P, Caraglia M, Abbruzzese A, Beninati S (2013). gamma-Tocopherol inhibits human prostate cancer cell proliferation by up-regulation of transglutaminase 2 and down-regulation of cyclins. Amino Acids.

[19] Kotsakis P, Wang Z, Collighan R, Griffin M (2011). The role of tissue transglutaminase (TG2) in regulating the tumour progression of the mouse colon carcinoma CT26. Amino Acids.

[20] Fok JY, Mehta K (2007). Tissue transglutaminase induces the release of apoptosis inducing factor and results in apoptotic death of pancreatic cancer cells. Apoptosis.

[21] Kotsakis P, Griffin M (2007). Tissue transglutaminase in tumour progression: friend or foe?. Amino Acids.

[22] Jones RA, Kotsakis P, Johnson TS, Chau DYS, Ali S, Melino G, Griffin M (2006). Matrix changes induced by transglutaminase 2 lead to inhibition of angiogenesis and tumor growth. Cell Death Differ.

[23] Hewitt RE, McMarlin A, Kleiner D, Wersto R, Martin P, Tsokos M, Stamp GW, Stetler-Stevenson WG (2000). Validation of a model of colon cancer progression. The Journal of pathology.

[24] Badarau E, Wang Z, Rathbone DL, Costanzi A, Thibault T, Murdoch CE, El Alaoui S, Bartkeviciute M, Griffin M (2015). Development of Potent and Selective Tissue Transglutaminase Inhibitors: Their Effect on TG2 Function and Application in Pathological Conditions. Chemistry & biology.

[25] Telci D, Collighan RJ, Basaga H, Griffin M Increased TG2 Expression Can Result in Induction of Transforming Growth Factor beta 1, Causing Increased Synthesis and Deposition of Matrix Proteins, Which Can Be Regulated by Nitric Oxide. Journal of Biological Chemistry;.

[26] Nyabam S, Wang Z, Thibault T, Oluseyi A, Basar R, Marshall L, Griffin M (2016). A novel regulatory role for tissue transglutaminase in epithelial-mesenchymal transition in cystic fibrosis. Biochim Biophys Acta.

[27] Volmer MW, Radacz Y, Hahn SA, Klein-Scory S, Stuhler K, Zapatka M, Schmiegel W, Meyer HE, Schwarte-Waldhoff I (2004). Tumor suppressor Smad4 mediates downregulation of the anti-adhesive invasion-promoting matricellular protein SPARC: Landscaping activity of Smad4 as revealed by a “secretome” analysis. Proteomics.

[28] Chow JYC, Cabral JA, Chang J, Carethers JM (2008). TGFβ modulates PTEN expression independently of SMAD signaling for growth proliferation in colon cancer cells. Cancer biology & therapy.

[29] Leppa S, Saffrich R, Ansorge W, Bohmann D (1998). Differential regulation of c-Jun by ERK and JNK during PC12 cell differentiation. Embo Journal.

[30] Clevers H, Nusse R (2012). Wnt/β-Catenin Signaling and Disease. Cell.

[31] Miyoshi N, Ishii H, Mimori K, Tanaka F, Hitora T, Tei M, Sekimoto M, Doki Y, Mori M (2010). TGM2 Is a Novel Marker for Prognosis and Therapeutic Target in Colorectal Cancer. Annals of Surgical Oncology.

[32] Yang LL, Liang CY, Lu TC, Zhi CY, Liu B, Zhou JH, Liu XM, Gao HC, Huang W (2013). Role of tissue transglutaminase and effect of cantharidinate in human colorectal cancer. Molecular Medicine Reports.

[33] Fernandez-Acenero MJ, Torres S, Garcia-Palmero I, Diaz Del Arco C, Casal JI (2016). Prognostic role of tissue transglutaminase 2 in colon carcinoma. Virchows Archiv.

[34] Mouradov D, Sloggett C, Jorissen RN, Love CG, Li S, Burgess AW, Arango D, Strausberg RL, Buchanan D, Wormald S, O’Connor L, Wilding JL, Bicknell D (2014). Colorectal Cancer Cell Lines Are Representative Models of the Main Molecular Subtypes of Primary Cancer. Cancer Research.

[35] Cao LY, Petrusca DN, Satpathy M, Nakshatri H, Petrache I, Matei D (2008). Tissue transglutaminase protects epithelial ovarian cancer cells from cisplatin-induced apoptosis by promoting cell survival signaling. Carcinogenesis.

[36] Mehta K, Kumar A, Kim HI (2010). Transglutaminase 2: A multi-tasking protein in the complex circuitry of inflammation and cancer. Biochemical Pharmacology.

[37] Kumar A, Xu J, Brady S, Gao H, Yu D, Reuben J, Mehta K (2010). Tissue Transglutaminase Promotes Drug Resistance and Invasion by Inducing Mesenchymal Transition in Mammary Epithelial Cells. Plos One.

[38] Fisher ML, Adhikary G, Xu W, Kerr C, Keillor JW, Eckert RL (2015). Type II transglutaminase stimulates epidermal cancer stem cell epithelial-mesenchymal transition. Oncotarget.

[39] Mehta K, Han A (2011). Tissue Transglutaminase (TG2)-Induced Inflammation in Initiation, Progression, and Pathogenesis of Pancreatic Cancer. Cancers.

[40] Neuzillet C, Tijeras-Raballand A, Cohen R, Cros J, Faivre S, Raymond E, de Gramont A (2015). Targeting the TGFβ pathway for cancer therapy. Pharmacology & Therapeutics.

[41] Olsen KC, Epa AP, Kulkarni AA, Kottmann RM, McCarthy CE, Johnson GV, Thatcher TH, Phipps RP, Sime PJ (2014). Inhibition of Transglutaminase 2, a Novel Target for Pulmonary Fibrosis, by Two Small Electrophilic Molecules. American Journal of Respiratory Cell and Molecular Biology.

[42] Ranganathan P, Agrawal A, Bhushan R, Chavalmane AK, Kalathur RKR, Takahashi T, Kondaiah P (2007). Expression profiling of genes regulated by TGF-beta: Differential regulation in normal and tumour cells. Bmc Genomics.

[43] de Caestecker MP, Piek E, Roberts AB (2000). Role of transforming growth factor-beta signaling in cancer. Journal of the National Cancer Institute.

[44] Morin PJ, Sparks AB, Korinek V, Barker N, Clevers H, Vogelstein B, Kinzler KW (1997). Activation of beta-catenin-Tcf signaling in colon cancer by mutations in beta-catenin or APC. Science.

[45] Condello S, Cao LY, Matei D (2013). Tissue transglutaminase regulates beta-catenin signaling through a c-Src-dependent mechanism. Faseb Journal.

[46] Ilyas M, Tomlinson IPM, Rowan A, Pignatelli M, Bodmer WF (1997). β-Catenin mutations in cell lines established from human colorectal cancers. Proceedings of the National Academy of Sciences of the United States of America.

[47] Myneni VD, Melino G, Kaartinen MT (2015). Transglutaminase 2-a novel inhibitor of adipogenesis. Cell Death Dis.

[48] Faverman L, Mikhaylova L, Malmquist J, Nurminskaya M (2008). Extracellular transglutaminase 2 activates beta-catenin signaling in calcifying vascular smooth muscle cells. Febs Letters.

[49] Chen SH, Chun YL, Lung TL, Geen DC, Pl Ping, Chin CH, Wen TK, Pei HT, Andrew VS, Jiuan JH, Ming TL (2010). Up-regulation of Fibronectin and Tissue Transglutaminase Promotes Cell Invasion Involving Increased Association with Integrin and MMP Expression in A431 Cells. Anticancer Research.

[50] Lee SH, Koo BS, Kim JM, Huang S, Rho YS, Bae WJ, Kang HJ, Kim YS, Moon JH, Lim YC (2014). Wnt/β-catenin signalling maintains self-renewal and tumourigenicity of head and neck squamous cell carcinoma stem-like cells by activating Oct4. The Journal of pathology.

[51] Kumar A, Gao H, Xu J, Reuben J, Yu D, Mehta K (2011). Evidence that aberrant expression of tissue transglutaminase promotes stem cell characteristics in mammary epithelial cells. Plos One.

[52] Fang L, Zhu Q, Neuenschwander M, Specker E, Wulf-Goldenberg A, Weis WI, von Kries JP, Birchmeier W (2016). A Small-Molecule Antagonist of the β-Catenin/TCF4 Interaction Blocks the Self-Renewal of Cancer Stem Cells and Suppresses Tumorigenesis. Cancer Research.

[53] Wang Z, Perez M, Caja S, Melino G, Johnson TS, Lindfors K, Griffin M (2013). A novel extracellular role for tissue transglutaminase in matrix-bound VEGF-mediated angiogenesis. Cell Death & Disease.

[54] Wang Z, Collighan RJ, Pytel K, Rathbone DL, Li XL, Griffin M (2012). Characterization of Heparin-binding Site of Tissue Transglutaminase its importance in cell surface targeting, matrix deposition, and cell signaling. Journal of Biological Chemistry.

[55] Akagi T, Sasai K, Hanafusa H (2003). Refractory nature of normal human diploid fibroblasts with respect to oncogene-mediated transformation. Proceedings of the National Academy of Sciences of the United States of America.

