# Supplementary material for: Tissue transglutaminase induces Epithelial-Mesenchymal-Transition and the acquisition of stem cell like characteristics in colorectal cancer cells
Source: Oncotarget. 2017 Feb 16;8(12):20025–41. doi: 10.18632/oncotarget.15370 (PMC5386741; doi:10.18632/oncotarget.15370)
Supplement: Supplementary file 1 [file oncotarget-08-20025-s001.pdf]

Tissue transglutaminase induces Epithelial-Mesenchymal-Transition and the acquisition of stem cell like characteristics in colorectal cancer cells

SUPPLEMENTARY FIGURES AND TABLE

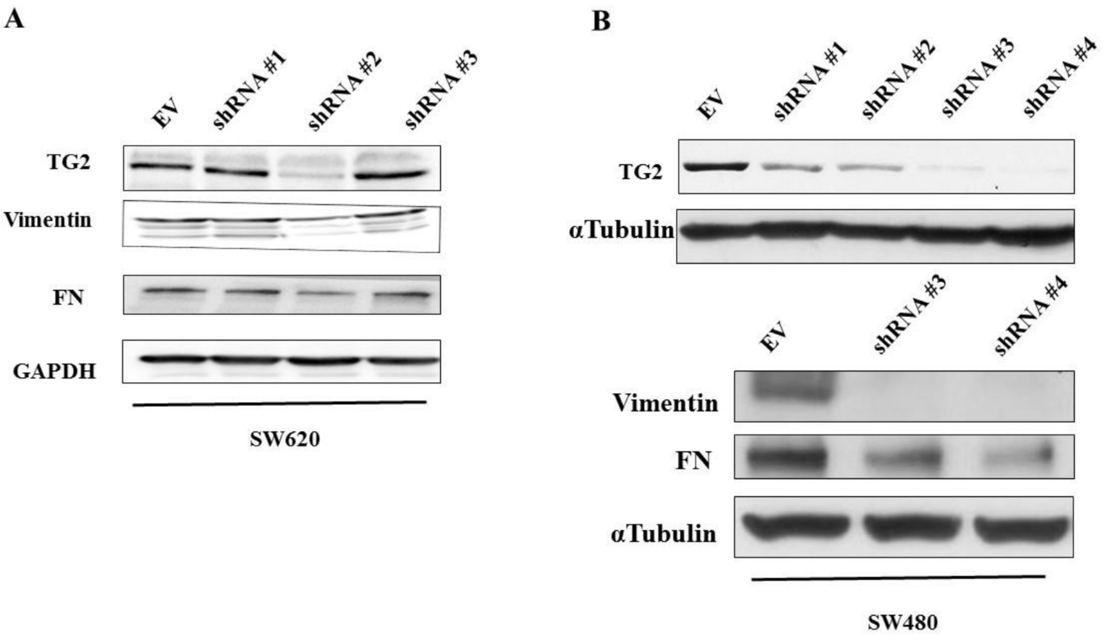

Supplementary Figure 1: TG2 knockdown to show efficiency of TG2 targeting shRNA. Western blot showing the effect of various TG2 shRNA constructs on TG2 and EMT markers (vimentin and fibronectin) with protein expression levels compared to the empty vector (EV) control cells in **A.** SW620 cells and **B.** SW480 cells.

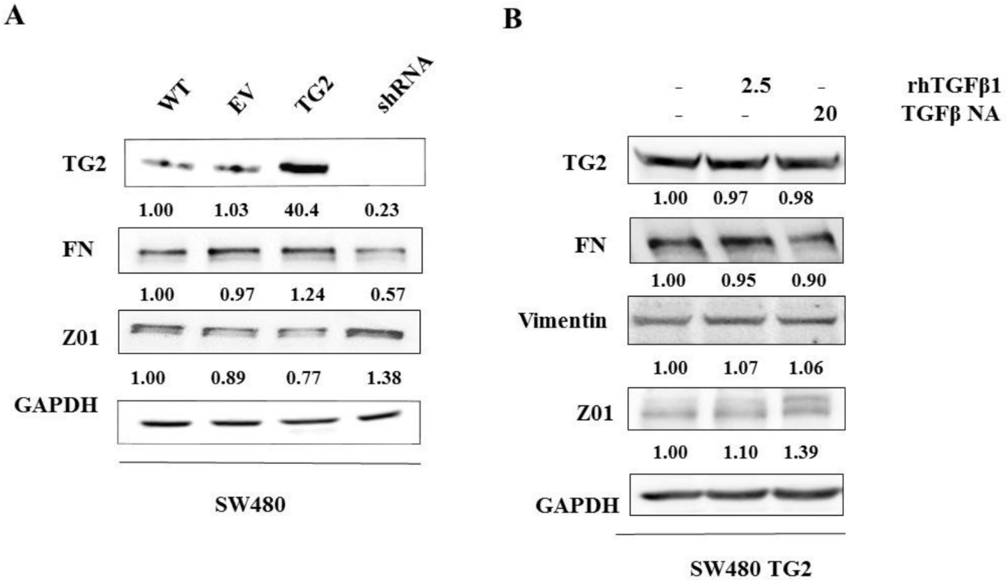

Supplementary Figure 2: TG2 knockdown to show efficiency of TG2 targeting shRNA. TGFβ1 does not significantly affect EMT in SW480 cells transduced with TG2. **A.** Western blot of TG2 and EMT markers (fibronectin and ZO1) in whole cell lysates from wt SW480 cells, SW480 cells transduced with empty vector (EV), human TG2 (TG2), and shRNA targeting TG2 (shRNA). Transduction of TG2 in SW480 cells has a slight but not significant impact on EMT compared to the control EV cells **B.** Western blot of whole cell lysates from SW480 cells transduced with TG2 (SW480 TG2) showing expression of TG2 and EMT markers (Fibronectin, vimentin and ZO1) with or without treatment with TGFβ1 or TGFβ neutralising antibody. CRCs were treated with human recombinant TGFβ1 (2.5 ng/ml) and TGFβ neutralising antibody (NA) (20 µg/ml) for 48 h.

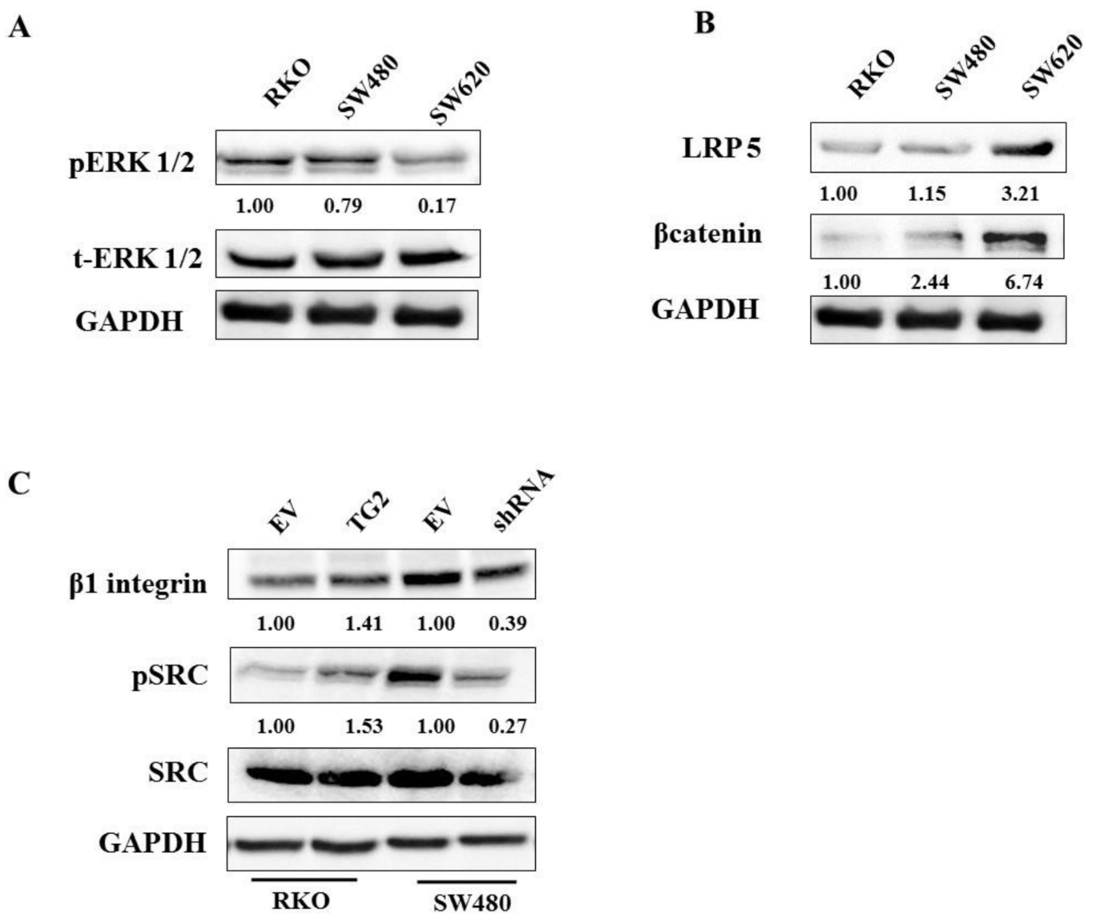

Supplementary Figure 3: TGFβ1 does not significantly affect EMT in wt SW480 cells and SW480 cells transduced with TG2. Characterisation of CRCs RKO, SW480 and SW620 for ERK1/2 , β-catenin and src signalling pathways. **A.** Western blotting showing reduced ERK1/2 activation in metastatic cell line SW620 compared to the two primary cancer cell lines RKO and SW480. **B.** Western blotting showing the characterisation of some of the elements of the β catenin signalling pathway. SW620 cells overexpress LRP5 (a receptor in the wnt/β catenin signalling pathway) and β catenin when compared to SW480 and RKO cells. **C.** Western blotting for β1 integrin expression and Src activation in CRCs with TG2 expression increased by viral transduction (RKO TG2) or reduced by transduction with TG2 shRNA (SW480shRNA) and their corresponding transduced empty vector (EV) controls. TG2 expression correlates with β1 integrin expression and Src activation.

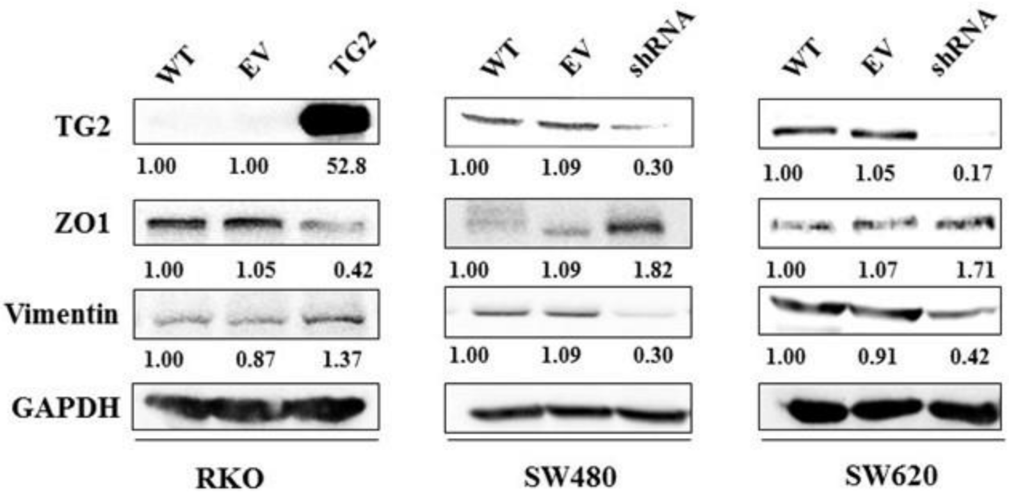

Supplementary Figure 4: TG2 and EMT markers are not affected by their transduction with empty control vector. Western blotting for EMT markers ZO1 and vimentin in whole cell lysates of wt cells, cells transduced with empty vector (EV, control cells) and cells transduced with lentiviral particles containing human TG2 (TG2) or shRNA targeting human TG2 (shRNA) in RKO, and SW480 and SW620 cells respectively.

Supplementary Table 1: Table showing a list of the primary antibodies used in the Methods with their commercial source.

Primary Antibody	Source	Specie
Twist	Santa Cruz Biotechnology Inc, UK	Rabbit
Slug	Santa Cruz Biotechnology Inc, UK	Rabbit
β-catenin	Santa Cruz Biotechnology Inc, UK	Mouse
p-Src(Y416)	Santa Cruz Biotechnology Inc, UK	Rabbit
Src	Santa Cruz Biotechnology Inc, UK	Rabbit
GAPDH	Santa Cruz Biotechnology Inc, UK	Rabbit
Vimentin	Santa Cruz Biotechnology Inc, UK	Mouse
αSMA	Santa Cruz Biotechnology Inc, UK	Rabbit
N cadherin	Santa Cruz Biotechnology Inc, UK	Rabbit
TGFβ receptor 1	Santa Cruz Biotechnology Inc, UK	Rabbit
TGFβ receptor II	Santa Cruz Biotechnology Inc, UK	Rabbit
p-Smad 3	Santa Cruz Biotechnology Inc, UK	Rabbit
t-Smad 2/3	Santa Cruz Biotechnology Inc, UK	Rabbit
t-ERK1/2	Santa Cruz Biotechnology Inc, UK	Mouse
p-ERK (T202/Y204)	Santa Cruz Biotechnology Inc, UK	Mouse
C-Jun	Santa Cruz Biotechnology Inc, UK	Rabbit
β1 Integrin	Santa Cruz Biotechnology Inc, UK	Rabbit
ZO-1	life Technologies, UK	Mouse
TG2	Neomark Pierce, UK	Mouse
Fibronectin	Sigma Aldrich, UK	Rabbit
